# Assessment of photodynamic therapy as a salvage treatment for local failure after chemoradiotherapy or radiotherapy for esophageal cancer in patients aged 80 years or older

**DOI:** 10.1002/deo2.167

**Published:** 2022-09-25

**Authors:** Michiko Nishikawa, Yoshinobu Yamamoto, Saeko Kushida, Taku Hirabayashi, Syunta Tanaka, Naoki Takegawa, Takuya Mimura, Hidetaka Tsumura, Ikuya Miki, Masahiro Tsuda

**Affiliations:** ^1^ Department of Gastroenterological Oncology Hyogo Cancer Center Hyogo Japan; ^2^ Department of Gastroenterology Kakogawa Central City Hospital Hyogo Japan

**Keywords:** aged, chemoradiotherapy, esophageal neoplasms, photochemotherapy, salvage therapy

## Abstract

**Objectives:**

Chemoradiotherapy (CRT) or radiotherapy (RT) alone is often the treatment of choice for elderly patients with esophageal cancer with the expectation of organ preservation. However, salvage treatment remains a problem when endoscopic resection is not indicated for local failure after CRT/RT. Photodynamic therapy (PDT) is indicated for local failure after CRT/RT, but there are few reports on its efficacy and safety in elderly patients. This study aimed to assess the outcome of PDT for local failure after CRT/RT for esophageal cancer in elderly patients.

**Methods:**

This retrospective single‐center study included 42 patients who first underwent PDT between April 2013 and June 2021. Patients aged ≥80 and <80 years were classified into the elderly and nonelderly groups, respectively. Local complete response rate, overall survival, progression‐free survival, and incidence of adverse events related to PDT were compared retrospectively between the groups.

**Results:**

The local complete response rate was 93.3% in the elderly group and 85.7 in the non‐elderly group. The 2‐year overall survival rate was 68.6% and 72.5%, and the 2‐year progression‐free survival rate was 49.5% and 70.0% in the elderly and nonelderly groups, respectively. There was no significant difference in any of these outcomes between the groups. In terms of adverse events, pneumonia and delirium tended to occur more frequently in the elderly group, but there were no serious adverse events in either group.

**Conclusion:**

The outcome of salvage PDT in the local control was comparable between the elderly and nonelderly patients for local failure after CRT/RT for esophageal cancer.

## INTRODUCTION

Esophageal cancer is the eighth most common cancer worldwide and the sixth leading cause of cancer‐related death.^1^ According to the Japanese guidelines for the treatment of esophageal cancer, clinically diagnosed T1a‐epithelial/lamina propria cancers are indicated for endoscopic resection, whereas esophagectomy or chemoradiotherapy (CRT) is the standard treatment for cancers with submucosal invasion.^2^ In elderly patients, CRT or radiotherapy (RT) alone is less invasive and is often the treatment of choice for organ preservation. A systematic review of patients aged ≥70 years with esophageal cancer reported that 37–64% underwent CRT, whereas 7–36% underwent surgery.^3^ However, salvage treatment was also necessary as the rate of local failure after CRT was as high as 30–40%.^4^, ^5^ For this reason, salvage treatment after CRT for esophageal cancer is a major problem in elderly patients.

Esophagectomy is a mostly curative but highly invasive treatment. It has been reported that compared with younger patients, in‐hospital mortality and the rates of incidence of cardiac and pulmonary complications after esophagectomy were higher in patients aged ≥80 years.^6^ In addition, salvage esophagectomy after CRT is associated with high rates of postoperative complications and mortality.^7^ Thus, salvage esophagectomy for locoregional failure after CRT carries a high risk in elderly patients.

In comparison, photodynamic therapy (PDT) is a less invasive endoscopic salvage treatment indicated for local recurrence and residual lesions after CRT or RT for esophageal cancer. An investigator‐initiated clinical trial of PDT that used a second‐generation photosensitizer, talaporfin sodium (Laserphyrin; Meiji Seika Pharma Co., Ltd., Tokyo, Japan), and a diode laser reported favorable treatment outcomes, with a local complete response (L‐CR) rate of 88.5% without serious adverse events.^8^ Based on the results of that study, salvage PDT using talaporfin sodium and a diode laser has been covered by insurance in Japan since 2015 for local failure after CRT for esophageal cancer.

However, the number of patients in the investigator‐initiated clinical trial was small, and the median patient age was 71.5 years, which is not an elderly population. With the aging of the population, the chances to treat elderly patients are increasing. Elderly patients have more comorbidities and tend to be in poor general condition than nonelderly patients. In some treatments, elderly patients cannot receive the benefits sufficiently because adverse events may increase. Although PDT is a less invasive treatment, there are few reports of the efficacy and safety of PDT focused on elderly patients. Therefore, the aim of this study was to assess the outcome of PDT using talaporfin sodium and a diode laser as a salvage treatment for esophageal cancer in elderly patients.

## METHODS

### Patients

This was a single‐center retrospective study. The subjects were 42 patients with esophageal cancer who first underwent PDT using talaporfin sodium between April 2013 and June 2021 at Hyogo Cancer Center, Hyogo, Japan. We classified patients aged ≥80 years into the elderly group and those aged <80 years into the nonelderly group. The inclusion criteria were as follows: (1) local recurrence or residual lesions within the irradiation field after CRT or RT for esophageal cancer, (2) invasion depth limited to clinical T1–T2 before PDT, (3) longitudinal lesion length within 3 cm and circumference of the lesion less than half that of the esophagus, (4) no invasion to the cervical esophagus, (5) absence of any lymph node or distant metastasis, (6) no indications for salvage surgery or patient refusal for salvage surgery, and (7) no indications for endoscopic resection. The exclusion criteria were as follows: (1) Eastern Cooperative Oncology Group Performance Status ≥3, (2) presence of other active cancers requiring surgery or chemotherapy, and (3) baseline lesions before CRT that involved the aorta.

This study was approved by the ethics board of Hyogo Cancer Center. The study procedures were carried out in accordance with the Declaration of Helsinki. Written informed consent was obtained from all patients.

### Staging

The clinical stage was determined using the tumor‐node‐metastasis classification of the International Union Against Cancer, 8th edition. The clinical T stage was evaluated by endoscopic observation using both white light and narrow band imaging and endoscopic ultrasonography, and the clinical N and M stages were evaluated by computed tomography.

### PDT procedure

Patients received 40 mg/m^2^ talaporfin sodium by intravenous administration, and the recurrent or residual lesion was then irradiated by diode laser at 100 J/cm^2^ with a fluence rate of 150 mW/cm^2^ within 4–6 h after administration. The next day, we performed endoscopic observation with additional laser irradiation when the residual tumor was observed or if the ischemic change was insufficient. All patients were instructed to stay in a room maintained at <500 lux for one week after administration of talaporfin sodium, and they were discharged after skin photosensitivity and other adverse events had disappeared. Patients were also instructed to avoid direct sun exposure for approximately one month after PDT.

### Follow‐up

We performed an endoscopic examination every 1–2 weeks after PDT. L‐CR was defined as follows: (1) disappearance of post‐PDT ulcers, (2) no residual tumor observed, and (3) disappearance of cancer cells by biopsy. If PDT was repeated for the same lesion because of residual tumor, the local response was calculated using the best response. We performed repeated PDT for local recurrence in patients once they have achieved L‐CR after PDT. Adverse events and toxicity were evaluated and graded according to the Common Terminology Criteria for Adverse Events version 5.0.

### Outcomes

The primary endpoint was the L‐CR rate for tumor lesions in the elderly and nonelderly groups. The secondary endpoints were overall survival (OS), progression‐free survival (PFS), and incidence of adverse events related to PDT in the two groups. OS was measured from the date of the first PDT to the date of death or the latest confirmation of survival. PFS was measured from the date of the first PDT to the detection of local failure or distant metastasis or the date of death or the latest confirmation of survival. When the curative treatment was possible for the recurrence of lesions by repeated PDT or endoscopic submucosal dissection (ESD), we did not define the lesions as a local failure.

### Statistical analysis

Categorical variables were expressed as the number and proportion, and compared using the Chi‐square test and Fisher's exact test. Continuous variables were expressed as medians and ranges, and compared using the Mann–Whitney *U*‐test. Survival time was calculated by the Kaplan–Meier method and compared by the log‐rank test. *p* < 0.05 was considered to be statistically significant. All statistical analyses were performed using SPSS version 27 (IBM Corp., Armonk, NY, USA) and EZR (Saitama Medical Center, Jichi Medical University, Saitama, Japan).

## RESULTS

### Patient characteristics

**FIGURE 1:**
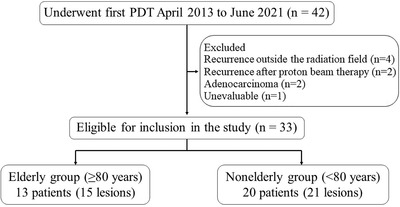
Flowchart of patient selection

There were 13 patients (15 lesions) in the elderly group and 20 patients (21 lesions) in the non‐elderly group (Figure 1). During the follow‐up of this study, eight patients underwent salvage surgery, and all of them were nonelderly (<80). Among these eight cases, only one case met the indication for PDT.

The patients’ baseline and clinical characteristics are listed in Table 1. The median age was 85.0 (range 80–91) and 70.5 (range 57–79) years in the elderly and nonelderly groups, respectively. In the patient background, there was no significant difference between the two groups for sex, Eastern Cooperative Oncology Group Performance Status, American Society of Anesthesiologists Performance Status, frequency of comorbidities, Charlson Comorbidity Index, and use of antithrombotic agents. There was also no significant difference between the two groups for the T stage and N stages before RT/CRT, the total dose of RT, tumor status after CRT/RT, or the interval between CRT/RT and PDT. The proportion of patients treated with RT alone was significantly higher in the elderly group than in the nonelderly group (53.8% vs. 15.0%, *p* = 0.026). There was no significant difference between the groups with regard to the location of the tumor before PDT, T stage before PDT, or total dose of irradiation. Median tumor size before PDT was significantly greater in the elderly group than in the nonelderly group (20 mm vs. 15 mm, *p* = 0.013).

**TABLE 1:** Baseline and clinical characteristics

	**Non‐elderly group (*n* [%]) (20 patients, 21 lesions)**	**Elderly group (*n* [%]) (13 patients, 15 lesions)**	***p‐*value**
**Age (median [range], years)**	**70.5 (57–79)**	**85.0 (80–91)**	
Sex			0.659
Male	17 (85.0)	10 (76.9)	
Female	3 (15.0)	3 (23.1)	
ECOG performance status			1.000
0–1	18 (90.0)	12 (92.3)	
2	2 (10.0)	1 (7.7)	
≥3	0 (0.0)	0 (0.0)	
ASA performance status			0.298
1	3 (15.0)	0 (0.0)	
2	12 (60.0)	8 (61.5)	
3	5 (25.0)	5 (38.5)	
≥4	0 (0.0)	0 (0.0)	
Comorbidities (with overlap)			
Hypertension	7 (35.0)	4 (30.8)	1.000
Diabetes mellitus	2 (10.0)	3 (23.1)	0.360
Cardiovascular disease	3 (15.0)	3 (23.1)	0.659
Cerebrovascular disease	2 (10.0)	1 (7.7)	1.000
Pulmonary disease	1 (5.0)	1 (7.7)	1.000
Charlson Comorbidity Index			0.502
0–2	13 (65.0)	7 (53.8)	
3–4	6 (30.0)	6 (46.2)	
≥5	1 (5.0)	0 (0.0)	
Use of antithrombotic agents	5 (25.0)	4 (30.8)	1.000
T stage before CRT/RT			0.286
T1	7 (35.0)	7 (53.8)	
T2	5 (25.0)	3 (23.1)	
T3	8 (40.0)	2 (15.4)	
T4	0 (0.0)	1 (7.7)	
N stage before CRT/RT			0.132
N0	11 (55.0)	11 (84.6)	
N1–	9 (45.0)	2 (15.4)	
Prior treatment			0.026
CRT	17 (85.0)	6 (46.2)	
RT alone	3 (15.0)	7 (53.8)	
Total dose of radiotherapy (Gy)			0.360
≥60	18 (90.0)	10 (76.9)	
<60	2 (10.0)	3 (23.1)	
Tumor status after CRT/RT			0.625
Recurrence	16 (80.0)	12 (92.3)	
Residual	4 (20.0)	1 (7.7)	
Interval between CRT/RT and PDT [median (range), months]	31.2 (4–90)	13.9 (3–183)	0.392
Location of the tumor before PDT			0.439
Ut	4 (19.0)	1 (6.7)	
Mt	13 (61.9)	11 (73.3)	
Lt	4 (19.0)	2 (13.3)	
Ae	0 (0.0)	1 (6.7)	
Tumor size [median (range), mm]	15.0 (10–25)	20.0 (7–30)	0.013
T stage before PDT			0.818
T1a	6 (28.6)	3 (20.0)	
T1b	13 (61.9)	10 (66.7)	
T2	2 (9.5)	2 (13.3)	
Total dose of irradiation [median (range), J]	350 (200–800)	500 (150–700)	0.214

Abbreviations: Ae, abdominal esophagus; ASA, American Society of Anesthesiologists CRT, chemoradiotherapy; ECOG, Eastern Cooperative Oncology Group; Lt, lower thoracic esophagus; Mt, middle thoracic esophagus; PDT, photodynamic therapy; RT, radiotherapy; Ut, upper thoracic esophagus.

### Outcome

The local efficacy of PDT is summarized in Table 2. The median follow‐up period was 21.0 months (range 4–86 months) and 22.8 months (range 7–98 months) in the elderly and nonelderly groups, respectively (*p* = 0.427). Of all lesions (36 lesions) in this study, L‐CR was achieved in 32 (the L‐CR rate: 88.8%). The L‐CR rate was 93.8% (30/32) in T1 before PDT and 50.0% (2/4) in T2. Of the 15 lesions in the elderly group, L‐CR was achieved in 14 (93.3%; 95% confidence interval [CI] 68.1–99.8%). Of the 21 lesions in the nonelderly group, L‐CR was achieved in 18 (85.7%; 95% CI 63.7–97.0%). There was no significant difference in the L‐CR rate between the groups (*p* = 0.626).

**TABLE 2:** Best response to photodynamic therapy (PDT)

	**Non‐elderly group (20 patients, 21 lesions)**	**Elderly group (13 patients, 15 lesions)**	***p‐*value**
L‐CR	18	14	
L‐nonCR	3	1	
L‐CR rate (95% CI)	85.7% (63.7–97.0)	93.3% (68.1–99.8)	0.626
Follow‐up period [median (range), months]	22.8 (7–98)	21.0 (4–86)	0.427

Abbreviations: CI, confidence interval; L‐CR, local complete response; L‐nonCR, local non‐complete response; PDT, photodynamic therapy.

**FIGURE 2:**
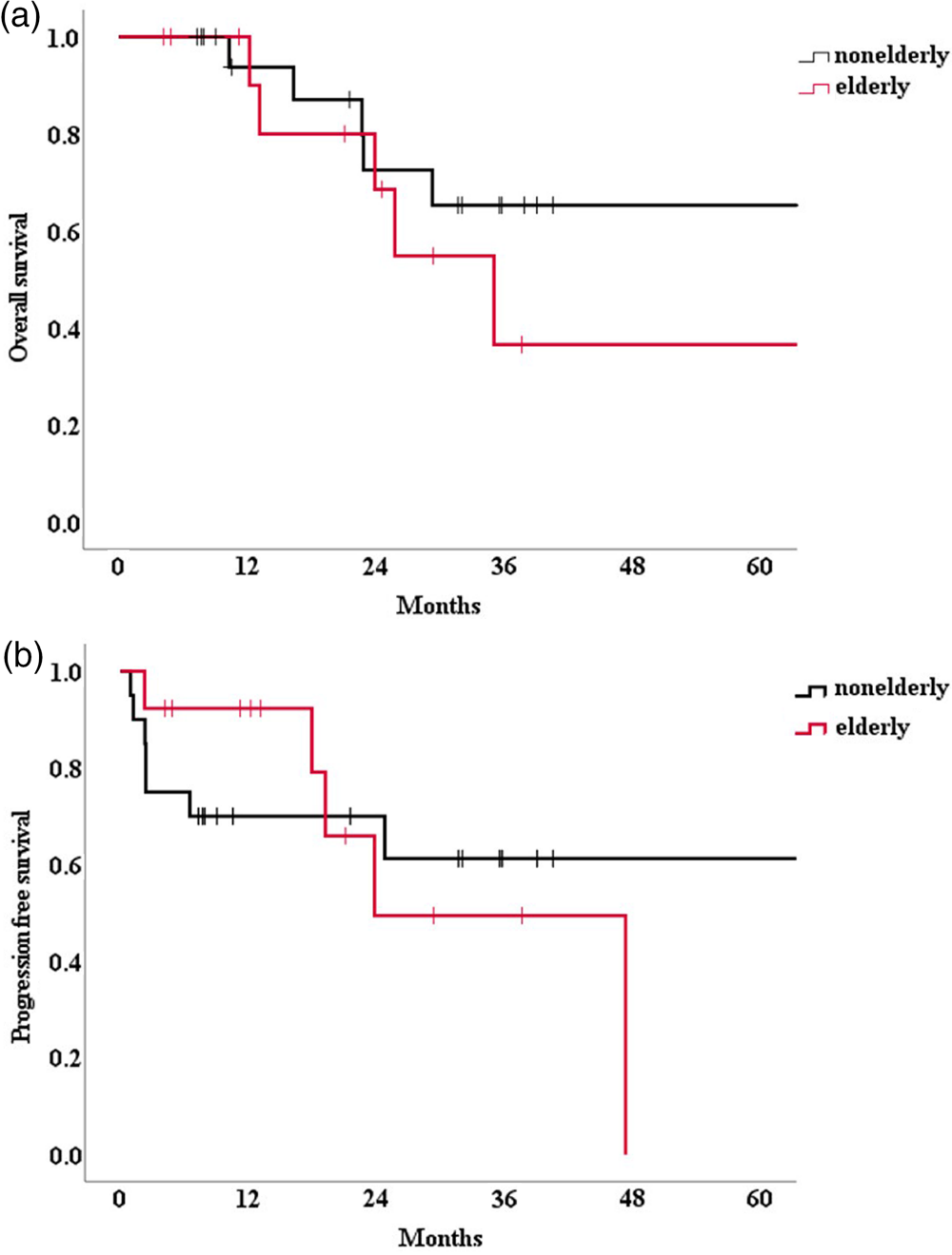
Overall survival and progression‐free survival. (a) Kaplan–Meier OS and (b) PFS curves after PDT. There was no significant difference in OS or PFS between the elderly and nonelderly groups. OS, overall survival; PFS, progression‐free survival; PDT, photodynamic therapy

The survival curves are shown in Figure 2. The 2‐year OS rate was 68.6% (95% CI 30.4–88.7%) and 72.5% (95% CI 42.1–88.8%) in the elderly and nonelderly groups, respectively (*p* = 0.380; log‐rank test). The 2‐year PFS rate was 49.5% (95% CI 12.9–78.5%) and 70.0% (95% CI 45.1–85.3%) in the elderly and nonelderly groups, respectively (*p* = 0.901; log‐rank test).

### Safety of PDT

Table 3 summarizes the details of adverse events related to PDT. Esophageal pain was significantly more frequent in the nonelderly group (70.0%; 14/20 patients) than in the elderly group (23.1%; 3/13 patients, *p* = 0.013), but all were grade 1. The rates of pneumonia (15.4%; 2/13 patients) and delirium (15.4%; 2/13 patients) were higher in the elderly group than in the nonelderly group (0%; 0/20) but did not reach statistical significance (*p* = 0.148). Grade 3 pneumonia was observed in one patient in the elderly group, the patients showed rapid improvement after fasting and administration of antibiotics. Esophageal hemorrhage, esophageal perforation, photosensitivity, and treatment‐related death were not observed in either group. Median hospital stay was 10.0 days (range, 9–31 days) and 10.5 days (range, 8–20 days) in the elderly and nonelderly groups, respectively (*p* = 0.676).

**TABLE 3:** Adverse events related to photodynamic therapy (PDT)

	**Non‐elderly group (*n* = 20)**					**Elderly group (*n* = 13)**					
	**Grade**					**Grade**					
	**1**	**2**	**3**	**4**	**Total (%)**	**1**	**2**	**3**	**4**	**Total (%)**	***p‐*value**
Esophageal pain	14	0	0	0	14 (70.0)	3	0	0	0	3 (23.1)	0.013
Esophageal stenosis	2	1	0	0	3 (15.0)	0	1	0	0	1 (7.7)	1.000
Fever	2	0	0	0	2 (10.0)	1	0	0	0	1 (7.7)	1.000
Pneumonia	0	0	0	0	0 (0.0)	0	1	1	0	2 (15.4)	0.148
Delirium	0	0	0	0	0 (0.0)	1	1	0	0	2 (15.4)	0.148
Esophageal hemorrhage	0	0	0	0	0 (0.0)	0	0	0	0	0 (0.0)	–
Esophageal perforation	0	0	0	0	0 (0.0)	0	0	0	0	0 (0.0)	–
Photosensitivity	0	0	0	0	0 (0.0)	0	0	0	0	0 (0.0)	–
Grade ≥3 adverse event					0 (0.0)					1 (7.7)	0.394

Abbreviation: PDT, photodynamic therapy.

## CLINICAL COURSE AFTER PDT

### Elderly group

**FIGURE 3:**
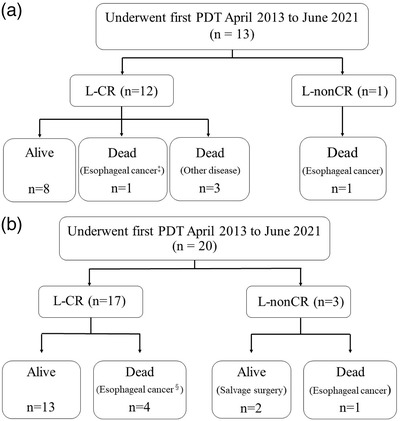
Clinical course in the elderly and nonelderly groups after PDT. (a) Elderly group. ^‡^ Recurrence of esophageal cancer at a different site to PDT PDT, photodynamic therapy; L‐CR, local complete response; L‐nonCR, local non‐complete response. (b) Non‐elderly group. ^§^Distant metastasis of esophageal cancer PDT, photodynamic therapy; L‐CR, local complete response; L‐nonCR, local non‐complete response

Of the 13 patients after PDT, 12 achieved L‐CR of all lesions, and eight are still alive. One died of local recurrence of esophageal cancer at a different site to that of PDT, and three died of diseases other than esophageal cancer. One patient with a local non‐complete response after PDT died of esophageal cancer (Figure 3a).

There were five patients with local recurrence after PDT, and four of them were treated with repeated PDT (second or third PDT), one of them was treated with ESD after repeated PDT.

**FIGURE 4:**
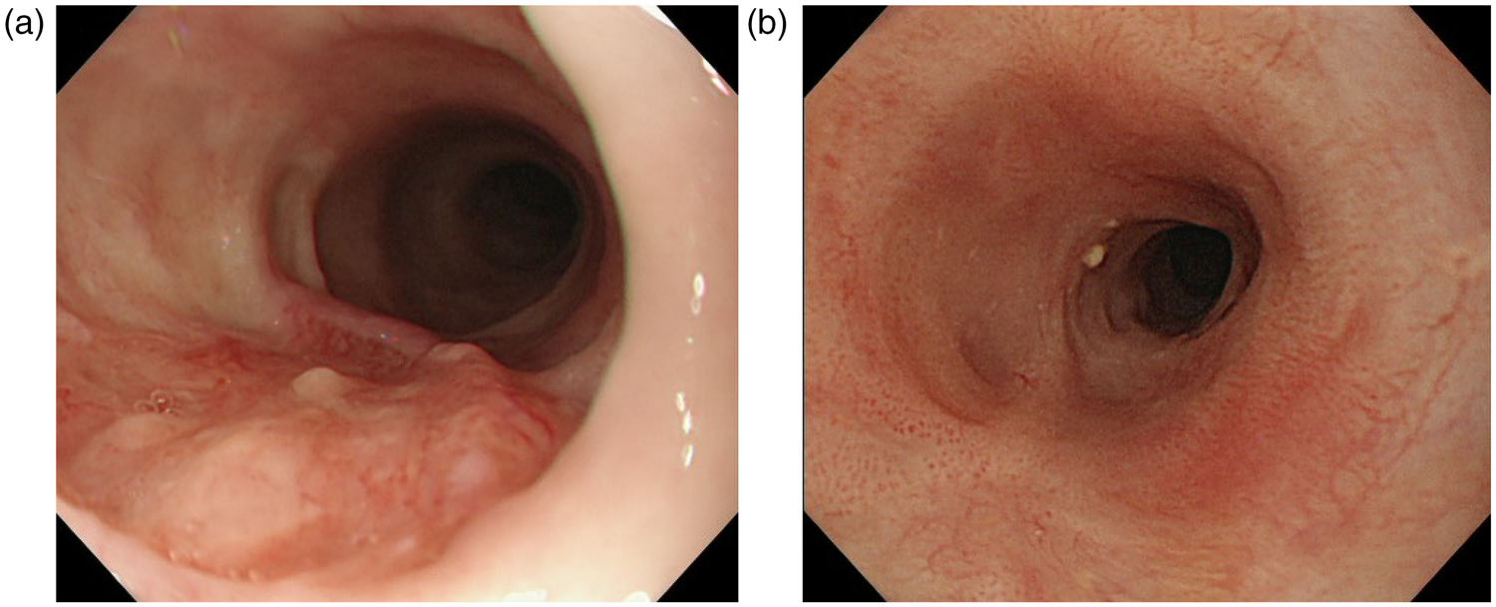
Endoscopic examination of a patient aged 80 years who achieved L‐CR after PDT. (a) Before PDT. A local recurrence lesion after chemoradiotherapy in the middle thoracic esophagus. The tumor depth was diagnosed as T2. (b) One year after PDT. There was no evidence of recurrence. PDT, photodynamic therapy; L‐CR, local complete response

A case of a patient aged 80 years who achieved L‐CR after salvage PDT (Figure 4).

### Nonelderly group

Of the 20 patients after PDT, 17 achieved L‐CR of all lesions, and 13 are still alive. Four died of distant metastasis of esophageal cancer (pleural metastasis, *n* = 1; lymph node metastasis, *n* = 2; lymph node and lung metastasis, *n* = 1). Of the three patients with local non‐complete response after PDT, two are still alive after salvage surgery and one died of esophageal cancer (Figure 3b).

There were three patients with local recurrence after PDT, and all of them were treated with repeated PDT (second or third PDT).

## DISCUSSION

This study evaluated the outcome of PDT after dividing eligible patients into elderly and nonelderly groups. In the elderly group, the L‐CR rate after PDT was 93.3%, which was as high as that in the non‐elderly group. In addition, there was no apparent increase in grade 3 or higher adverse events compared with the nonelderly group. To the best of our knowledge, this is the first study to assess the outcome of PDT in elderly patients.

L‐CR rates after PDT have been reported as 69–87.1% in single‐center studies.^9^, ^10^, ^11^, ^12^ Amanuma et al. reported a 2‐year OS rate after PDT of 66%, and a 2‐year PFS rate of 32%.^10^ In previous studies, the T stage before CRT, the T stage before PDT, and N0 before CRT were factors related to the outcome after PDT.^13^, ^14^ In the present study, there were almost no differences in these factors between the elderly and nonelderly groups, and the L‐CR and survival rates in the elderly group could be regarded as equivalent to those in previous reports. In this study, despite the high L‐CR rate, PFS was low in either group, especially in the elderly group. These findings could be due to cases with distant metastasis in either group and cases with local recurrence of esophageal cancer or death from other than esophageal cancer in the elderly group.

The ulcers after PDT are often deeper than the ulcers after ESD. Salvage ESD after PDT is technically more challenging because of severe fibrosis. Therefore, we performed repeated PDT for the local recurrence after PDT. Almost all the lesions could be cured by repeated PDT, but we performed ESD for local recurrence after repeated PDT in one case.

In the present study, the proportion of patients treated with RT alone was significantly higher in the elderly group than in the non‐elderly group. According to the Comprehensive Registry of Esophageal Cancer in Japan from 2009 to 2011 of the Japan Esophageal Society which included 2352 patients with esophageal cancer treated by RT alone or by CRT, the proportion of elderly patients was significantly higher in the RT group than in the CRT group.^15^ Some previous studies evaluated the efficacy of RT and CRT in elderly patients. Several studies have reported that RT alone has a poor prognosis compared with CRT. Jingu et al. reported that in the patients aged 80 years who were treated with RT alone with cStageⅡ and III disease, the prognosis was significantly worse for RT alone compared with CRT.^16^ Furthermore, it was reported that 40% of patients who received RT had residual lesions, and an additional 24% had local recurrence.^4^ However, chemotherapy is not suitable for many elderly patients due to organ dysfunction, and it is therefore important to consider salvaging PDT for elderly patients with esophageal cancer who have local failure following RT alone. Moreover, all patients who underwent salvage surgery at our institution were nonelderly. These results suggest that salvage surgery might be discouraged in elderly patients. This study revealed that the L‐CR rate after PDT was high and the survival rate was relatively favorable. These findings suggest that salvage PDT, which is a minimally invasive local treatment, may improve prognosis even in elderly patients who have poor tolerance for chemotherapy and surgery.

In terms of adverse events related to PDT using talaporfin sodium, previous studies have reported esophageal stenosis (4.5–41.7%^9^, ^10^, ^11^, ^12^, ^13^, ^17^), esophageal pain (43.5–54.8%^8^, ^12^, ^17^, ^18^), and fever (12.9−30.8%^8^, ^12^, ^17^, ^18^). Compared with those results, the incidence of adverse events was the same or lower in the present study, even in the elderly group. Following endoscopic treatment, the incidence of pneumonia after gastric ESD was reported as 2% in patients aged > 85 years,^19^ and that after esophageal ESD was 0% in patients aged > 80 years and 0.3% in patients under < 80 years, and the incidence of pneumonia was not significantly different.^20^ Although pneumonia was observed in two of the present patients (15.4% in the elderly group), one of whom was grade 3, the patient was treated successfully and did not develop severe progression of the disease. Hence, the rates of pneumonia and delirium tended to be high in the present elderly group, but there were no serious adverse events. Taken together, the findings of this study indicate that PDT is a safe and well‐tolerated treatment for elderly patients.

There are several limitations to this study. First, it is a single‐center, retrospective study. Second, the sample size was small and the follow‐up period was limited, so it is necessary to confirm the efficacy of PDT in elderly patients in a larger number of patients and with longer follow‐up periods. However, the safety of PDT for elderly patients could be demonstrated even in a small number of cases. Third, there were several selection biases in this study. The median tumor size before PDT was significantly greater in the elderly group. However, there was no significant difference in the L‐CR rate in the two groups, while adverse events did not increase in the elderly group. In addition, the proportion of residual lesions before PDT was higher in the nonelderly group than in the elderly group. This difference may have affected the L‐CR rate.

In conclusion, the outcome of salvage PDT in the local control was comparable between the elderly and nonelderly patients with local failure after CRT/RT for esophageal cancer, and salvage PDT might be a safe and well‐tolerated treatment for the elderly.

## CONFLICT OF INTEREST

None.

## FUNDING INFORMATION

None.
